# Supplementary material for: Inclusion of photoautotrophic cultivated diatom biomass in salmon feed can deter lice
Source: PLoS One. 2021 Jul 29;16(7):e0255370. doi: 10.1371/journal.pone.0255370 (PMC8321285; doi:10.1371/journal.pone.0255370)
Supplement: S1 Appendix — (DOCX) [file pone.0255370.s001.docx]

**S1 Appendix. Solvent gradient. Solvent gradient program used for normal-phase liquid chromatography of lipid classes, mobile phase A = isooctane/ethyl acetate (99.8:02); Mobile phase B = acetone/ethyl acetate (2:1 v/v) and 0.15% acetic acid; Mobile phase C = isopropanol/H_2_O (85:15 v/v).**

| Time (min) | Solvent A | Solvent B | Solvent C | Flow (ml min^-1)^ | Curve |
| --- | --- | --- | --- | --- | --- |
| 0.0 | 100 | 0 | 0 | 1.5 | 1 |
| 1.5 | 100 | 0 | 0 | 1.5 | 6 |
| 1.6 | 97 | 3 | 0 | 1.5 | 6 |
| 6.0 | 94 | 6 | 0 | 1.5 | 6 |
| 8.0 | 50 | 50 | 0 | 1.5 | 6 |
| 8.1 | 46 | 39 | 15 | 1.5 | 6 |
| 14.0 | 43 | 30 | 27 | 1.5 | 6 |
| 14.1 | 43 | 30 | 27 | 1.5 | 6 |
| 18.0 | 40 | 0 | 60 | 1.5 | 6 |
| 23.0 | 40 | 0 | 60 | 1.5 | 6 |
| 24.0 | 0 | 100 | 0 | 1.5 | 6 |
| 25.0 | 0 | 100 | 0 | 2.0 | 6 |
| 27.0 | 0 | 100 | 0 | 2.0 | 6 |
| 27.1 | 100 | 0 | 0 | 2.0 | 6 |
| 36.0 | 100 | 0 | 0 | 2.0 | 6 |
