# Supplementary material for: Inclusion of photoautotrophic cultivated diatom biomass in salmon feed can deter lice
Source: PLoS One. 2021 Jul 29;16(7):e0255370. doi: 10.1371/journal.pone.0255370 (PMC8321285; doi:10.1371/journal.pone.0255370)
Supplement: Attachment — Submitted filename: Response to Reviewers PONE-D-21-08390.docx [file pone.0255370.s008.docx]

To the Academic editor, PLOS ONE

Tzong-Yueh Chen

UiT The arctic university of Norway

I hereby submit a revised version of the manuscript no. PONE-D-21-08390 with title “Inclusion of photoautotrophic cultivated diatom biomass in salmon feed deters lice” (new title: “Inclusion of photoautotrophic cultivated diatom biomass in salmon can deter lice”. We have changed the manuscript as follows, according to the points raised by the academic editor and reviewer, plus some other issues. Our response and changes are:

| Academic editor comments: | Responses |
| --- | --- |
| **1**. The data cannot support the conclusions | I guess here is meant that we failed to detect an active ingredient/compound that could explain the reduction in lice infestation. In the original version it was stressed that so was the case, and that the only thing that differed significantly was FFA that was present at higher concentrations in feed with algae (and Calanus). We though feel that it was correct, at this stage, not to attribute this to lice reduction! We have though changed the Conclusion to be more “balanced” (see further below), and the title to “can deter lice” from “deters lice”, plus several changes in the manuscript itself. |
| **2-5**: -Change ethics Statement to include information from Methods section;  -Please upload a copy of Figures 3 and 4, to which you refer in your text on page 22. If the figure is no longer to be included ….. | -Ethics statement included  -No competing interest statement fixed  -Figures 3 and 4 were removed from manuscript, all references to them are now removed! |
| Reviewers comments:  **Reviewer #1:** |  |
| 1. How about the dose-dependent manner of the diatom biomass in the present study? | This was a pilot study with limited amounts of microalgae (diatom) biomass available. The aim was therefore to monitor growth and lice infestation vs. chemical agents represented by feed with different origins (fish oil, Calanus oil, standard feed) rather than different amounts of diatom biomass. But, an experiment underway will be aimed at examining this, i.e. different amounts of diatom biomass (with all other constituents and treatments kept constant). |
| 2,3 = Remarks that are not questions, from referee |  |
| **Reviewer #2:** |  |
| 1. The language of this manuscript should be properly checked by English native speaker. The content in the "Introduction" is a bit too long with redundant sentences. | Language is checked and Introduction has been shortened! See file «Revised Manuscript with Track Changes” |
| 2. "A" to "An" on line 36. | Fixed |
| 3. "long-chain polyunsaturated fatty acids (LC-PUFA)" to "LC-PUFA" on line 72. | Fixed |
| 4. What is "temperature areas" in line 102? | It is written temperate (not temperature) in line 102. Temperate area is the geographical area 40 – 60^o^N (and 40-60^o^S). |
| 5. Please modify paragraph in lines 107-111. | Paragraph is re-written to “Since salmon lice is a copepod, we hypothesized that inclusion of diatoms in the fish feed could reduce lice infestation. To test this, we offered four diets of different origins to salmon, including one with diatoms, and monitored lice infestation”. |
| 6. The range between 50-70000L is quite huge (lines 125-126), please make sure on this description. | Oops, shall be 50 000L – 70 000L, has been changed |
| 7. Please describe how the standard protocol of exposure was done (lines 169-170) | More detailed explanation now added, i.e: “Infection of the salmon was performed using a challenge model developed with the Aquaculture Research Station in Tromsø, approved by The Norwegian Food Safety Authority (ref. id 7509). This is a bath challenge model, where the fish is challenged with a known number of lice copepodids in stagnant seawater. The research station routinely produces infective copepodids by performing controlled infections, harvesting mature eggs and incubating these until they reach the copepodid stage. The dose of infection was calculated on the basis of an expected average estimate of 10 lice per fish.  The experiments were performed in the salmon louse laboratory at the research station, where factors such as water temperature are closely monitored and controlled. The water outlet from the laboratory is disinfected to prevent distribution of live copepodids to the environment. For more specific description of the model see [New ref, Skjelvareid et al., 2018]». |
| 8. Line 177, "try" to "dry". | Fixed ! |
| 9. Line 216, "International" to "international". | It was Internal, not international, but fixed from Internal to internal. |
| 10. Please use dot (.) not comma (,) for decimal point through the whole manuscript. | Have checked and changed this! |
| 11. The format of describing company (e.g. see lines 242 and 254 on page 11 for Sigma Aldrich). | Have fixed! |
| 12. Line 295, "Statment" to"Statement". | Fixed! |
| 13. The description on lines 311-312 is wrong (e.g. fish fed with diet A and D was significant higher than fish that fed with diet B and C for T1 to T2), please correct it. | It has been changed to the correct statement: “Fish fed diets A and D had overall significantly higher SGR (nested ANOVA, p < 0.001) than fish fed diet B and C for T0 to T3, i.e. for whole period, days 0 – 67) (Table 2, Fig 1)…. |
| 14. Please provide the content of carbohydrate in Table 3. | Carbohydrate was not measured, this since it is normally not done here defining food quality. However, if capacity/competence had been present it should possibly have been included. |
| 15. The description on lines 366-367 is wrong, please correct it. | “Oops, changed to: “Diet A (Algae) and C (Calanus) were identical with respect to amino acid content, except for glutamic acid (Glu) that was significantly lower in diet A than in the other diets” |
| 16. Line 369, "Tyrosine" to "tyrosine". | Fixed! |
| 17. Line 405, "EEA" to "EAA". | Fixed! |
| 18. Line 422, "were" to "higher" (I am not sure)? | Corrected to: “Fish fed diet A and B were higher in in omega-3 PUFAs (EPA + DHA) compared to the other diets. Diet C resulted in the lowest amounts of omega-3 PUFAs, i.e. less than half of the content in fish fed diets A and B”. |
| 19. Line 453, "[42] [43,44]" to "[42-44]". | Fixed! |
| 20. Lines 455-458. The authors should not summarize higher growth in this manuscript compared to other previous studies is due to good health. Does that mean the fish in other studies are all in bad health? | Ok, we “admit” this shall be toned down, and have changed the actual paragraph to: “Our fish hence performed in the high end of obtainable growth for salmon at the same weight and temperature [46]. The SGR range between the four diet groups also varied little (1.29 to 1.44 % day^-1^), and we interpret this as an indication of that the fish were at acceptable health compared to what is common in salmon aquaculture”. |
| 21. Lines 488 and 490, I can't see Fig.3 and Fig. 4. Are they suppose to be Table 5 and Table 8, respectively? | Correct, this has been changed!! |
| 22. What is "though stands" (Line 544)?. | This is in the Conclusion section, that now has been changed to: “Our investigation showed statistically significantly reduced lice infestation in salmon offered conventional feed with diatom biomass. In our investigation there were no correlations between feed ingredients and lice infestation. Neither were we able to detect a bioactive compound that was active as a lice deterrent, nor could we relate differences in lice infestation to health differences. The main result that lice infestation was reduced in the salmon group offered diatom biomass though still indicates the presence of some active lice deterring ingredient. In addition to that the ingredient may come from the feed, it can also have been synthesized or resulted from chemical reactions in the fish. We therefore strongly believe that further investigations should be undertaken in order to get more insight into the potential production and secretion of invertebrate repelling aldehydes in salmon, and that experiments should also take place in open sea pens under natural conditions”. |
| 23. Line 545, "healt" to "health". | Ok! |
| 24. Please organize the format for "Reference". | Done! |
| 25. The language in supplementary data should switch to English | Ok, done! |

22.05.2021

Hans Chr. Eilertsen (sign.)
