# Supplementary material for: Inclusion of photoautotrophic cultivated diatom biomass in salmon feed can deter lice
Source: PLoS One. 2021 Jul 29;16(7):e0255370. doi: 10.1371/journal.pone.0255370 (PMC8321285; doi:10.1371/journal.pone.0255370)
Supplement: Attachment — Submitted filename: Response to Reviewers.docx [file pone.0255370.s009.docx]

| **Reviewers comments** | **Author responses** |
| --- | --- |
| **Reviewer #1:** . |  |
| The authors did not respond to the two remarks "2. The potential indicator or the active compound for reducing lice in diatom were necessary to achieve the reproducibility in the present study." and "3. The authors assessed the composition in diets and salmon, and those results were further used to analyze the correlation to lice infestation. Unfortunately, there was no direct linkage between the decreased lice infestation and diatom.". These were not questions, but comments. | We understand that these points were comments but will anyway answer/comment to these points because they points to important issues in the manuscript. Our statements here are that we, before this investigation was performed, had a hypothesis that there was some ingredient (here oxylipin) that could cause reduced lice infestation. We though were unable to detect such a compound. The question is then what caused this, and our answer here is that it obviously was not some general health issues related to poor growth or feed ingredient. What was exclusive for the salmon that experienced reduced lice was feed with algae inclusion. We therefore only can give “circumstantial” evidence that it was something in the algae that caused this directly or that it was algae ingredients that triggered production of e.g. an aldehyde in the salmon. We have tried to even clearer in the abstract and conclusion here. Now we state in abstract: “*What was exclusive for salmon that experienced reduced lice was diatom inclusion in the feed. This therefore still indicates the presence of some lice deterring ingredient, either in the feed, or an ingredient can have triggered production of an deterrent in the fish*”. |
| **Reviewer #2:** |  |
| 1. In the introduction the authors stated that “oxylipin” is a group of copepod grazing deterrents in marine ecosystems. But why the authors did not detect oxylipin in the algae, feed or fish fillets? | See comment (to reviewer 1) above, i.e. we were not able to detect some active substance (oxylipin, group aldehyde), but it may be due to that such ingredients are unstable. The results therefore do not exclude that some deterrent was active, but the only “hard evidence” was the presence of diatoms in the feed that caused reduced lice infestation. We as mentioned above have made this clearer both in abstract and conclusion! |
| 2. The inclusion of marine oil in Norwegian fish feed has the resent years decreased to “The inclusion of marine oil in Norwegian fish feed in the recent years has decreased” in line 59. | This has been fixed! |
| 3. In line 69, “affect” to “effect”. | This has been fixed! |
| 4. Line 80, reference is required. | References no. 1 and 8 should be here, has been added! |
| 5. Line 477, Fish fed diet A and B were higher in in omega-3 PUFAs (EPA + DHA) compared to the other diets. However, in Table 8, the amount of EPA+DHA was group B (0.64) > group A (0.50) > group D (0.40) > group C (0.19). Therefor this statement is wrong. | Changed to: «Fish fed diet B were highest in omega-3 PUFAs (EPA + DHA), i.e. 0.64g/100g flesh, followed by diet A (0.50), D (0.40) and C (0.19)”. |
| 6. Line 479, “i.e. less than half of the content in” seems an unfinished sentence. | Changed to: « Diet C resulted in the lowest amounts of omega-3 PUFAs, i.e. less than half of the content in the other treatments”. |
| ------------------------------- |  |
| Else there are some other minor linguistic changes implemented |  |
| Ethics statement appears two places! | 26.06.21… removed Ethics statement from initial fish experiment description and ensured it is only in Methods under separate heading! |

26.06.2021

Hans Chr. Eilertsen (sign.)
